# A novel approach in assessment of root canal curvature

**Published:** 2009-10-10

**Authors:** Shiva Sadeghi, Vahideh Poryousef

**Affiliations:** 1 Department of Endodontics, Dental School/Dental Research Center, Guilan University of Medical Sciences, Rasht, Iran; 2 Private Practice, Rasht, Iran

**Keywords:** Endodontics, Root canal preparation, Root canal therapy.

## Abstract

Introduction: The purpose of this *in vitro* study was to introduce a new method to describe root canal curvatures and to assess the degree of curvature of human permanent mandibular teeth with curved root canals.

Materials and Methods: One hundred and thirty five mesial root canals of mandibular first and second molar teeth were selected. Access cavities were prepared. After inserting a K-file size #10 into each canal, radiographs were taken. Canal curvature was determined by measuring the Schneider angle, canal access angle, as well as the canal radius, length, height and curvature starting distance on scanned radiographs using a computerized image processing system. Data was evaluated statistically using Pearson correlation.

Results: The mean canal access angle (CAA) and Schneider angle (S) were 8.04^◦ ^(3.46) and 19^◦ ^(6.99), respectively. The Pearson correlation analysis found significant positive correlation between S and CAA (r=0.826, P<0.0001). Negative correlations were found between radius and length (r= –0.4, P<0.0001), radius and Schneider angle (r= –0.4, P<0.0001), radius and CAA (r= –0.24, P=0.004) and CAA and curvature starting distance (r= 0.4, P<0.0001). There was no correlation between height and distance (r=0.013, P=0.789), as well as CAA and height (r=0.654, P=0.001).

Conclusion: Under the limitations of this study, the results indicated that the shape of root canal curvature can be more accurately described using two angles, Schneider in combination with Canal access angle. The related parameters included radius, length, distance and height of curvature. [Iranian Endodontic Journal 2009;4(4):131-4]

## Introduction

Endodontic treatment consists of different stages including access cavity preparation, instrumentation, irrigation, and obturation of the root canal system, which can all be influenced by root canal curvature. Several studies were conducted to describe canal curvatures (1-5). The most common method to describe canal curvature was published by Schneider (6,7) in which an arbitrary angle was used as the only parameter. Pruett *et al.* pointed out that two canals which have the same angle as measured by the Schneider method could have very different radii or abruptness of curvature; and therefore have different levels of instrumentation difficulty. The authors suggested that measuring the angle of curvature, according to Schneider method, in combination with the radius of the curve is by far a better method for describing the canal curvature (6,8).

Schafer *et al.* investigated the frequency and degree of canal curvatures in human permanent teeth using both the Schneider angle (S) and the radius of curvature. They reported that the angle, the radius, and the length of the curve should be given in order to define the canal curvature mathematically and precisely (6).

In 2005, Gunday *et al.* introduced the term “canal access angle” (CAA) and two new curvature parameters pertaining to the coronal zone of curved root canals: the curvature starting distance (y) and the curvature height (x). They compared the new technique with Schneider method and reported that CAA is a more effective way of evaluating the root canal curvature (9). 

**Figure 1:**
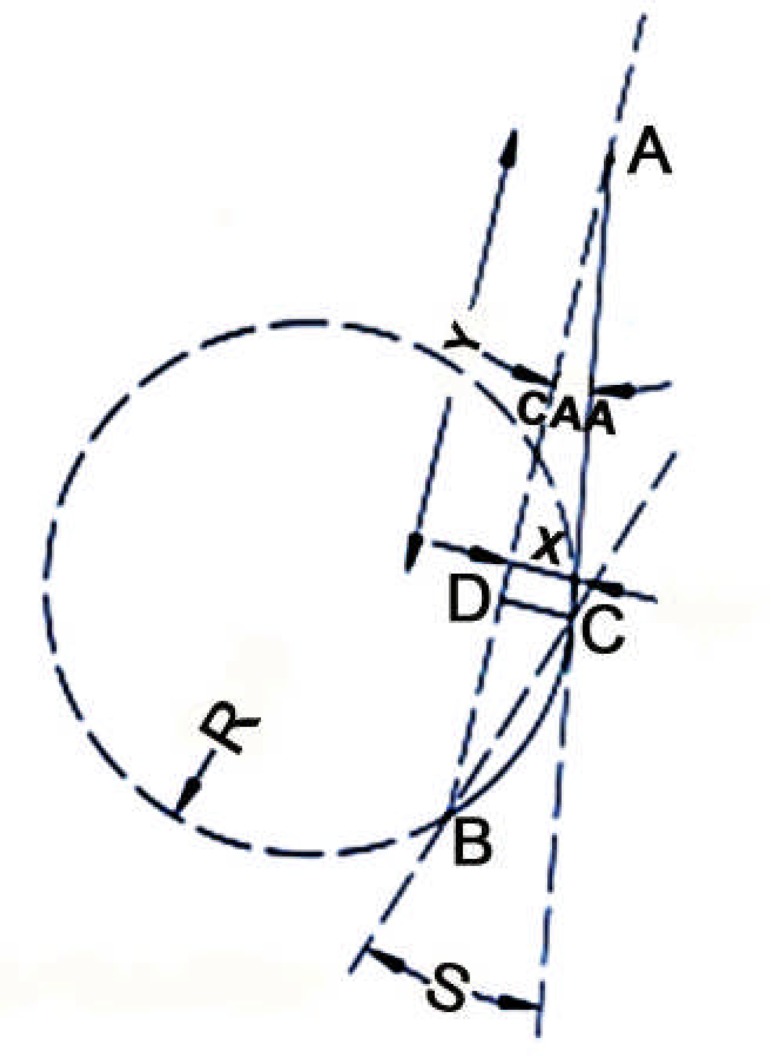
A, Canal orifice; B, apex; C, the point that the canal begins to move away from the long axis; D, the point that the perpendicular line from C intersects line AB; Canal access angle (CAA), the angle between line AB and line AC; Schneider angle (S), the acute angle between line BC and line AC; CD (X), canal height; AD (Y), canal distance; R, radius

Schneider angle, when used in combination with the radius and length of the curve, may provide a more precise method for describing the apical geometry of canal curvature while CAA and its related parameters may provide more information about the coronal geometry of canal curvature (9). 

The purpose of this study was to introduce a new method to determine the degree of curvature of human permanent mandibular teeth with curved canals.

## Materials and Methods

One hundred and thirty five mesiobuccal and mesiolingual canals of recently extracted permanent human mandibular first and second molars were used in this *in vitro* study. Before experimental procedures, all teeth were stored in normal saline and disinfected with 5.25% hypochlorite solution for 30 minutes. Following assessment for complete root formation, soft tissue and calculus were removed mechanically. After access cavity preparation, a K-flexofile size #10 (Dentsply Maillefer, Ballaigues, Switzerland) was placed in each canal extending to the apical foramen and radiographs were taken. The teeth were placed in a wax mould which was attached to a Kodak Ultra-speed film (Kodak, Stuttgart, Germany) and were aligned so that the long axis of the root canal was parallel and as near as possible to the surface of the film. The X-ray tube, and thus the central X-ray beam, was perpendicular to the root canal. The exposure time (0.40 s; 70 kv, 8 mA), source-to-film distance (27 cm) and object-to-film distance (5 mm) was kept constant for all radiographs. The films were developed, fixed and dried in unchanging and standard conditions.

The radiographs were scanned (Scanner: Agfa-Duascan, Germany) and all the angular and linear values were plotted using the program Auto CAD 2008. The Schneider angle consists of tracing a straight line (AC) from the orifice (point A) along the K-file in the coronal portion of the canal; this line is parallel to the long axis of the canal. A second straight line (BC) is drawn from the apex (point B) until it meets the first line (AC) at a point (C) where the canal lumen begins to move away from the long axis. This acute angle is measured and called the Schneider angle. The line between points B and C is a chord of the hypothetical circle that defines the curved part of the canal. The curved part of the root canal between points B and C is the arc of the hypothetical circle, which is specified by its radius. The radius can be calculated on the basis of the measured length of the cord between points B and C. Thus, the radius and length of curvature can be calculated according to the following formulas (6): 

*Radius *(*R*) = *BC/*2sin*S*, 

*Length *(*L*) = 4π*S*/360.

The CAA involves connecting the canal orifice (A) and apex (B) points with a line. The angle formed by the intersection between line (AB) and one parallel to the long axis of the canal from the coronal part (AC) is defined as the CAA. At the point C, a perpendicular line was drawn to AB. The point that the perpendicular line intersects AB is D. CD gives the curvature height, and the distance from A to point D is the curvature distance (AD=y) (9) (Figure 1).

**Table 1:** Mean and standard deviation (SD) of different curvature parameters (n=135)

Parameter	Mean	SD
Schneider angle (˚)	**19.15**	**6.99**
Canal access angle (˚)	**8.04**	**3.46**
Curvature radius (mm)	**19.1**	**1.03**
Curvature length (mm)	**6.6**	**0.24**
Curvature height (mm)	**1.8**	**0.078**
Curvature distance (mm)	**13.2**	**0.22**

Collected data were evaluated statistically using Pearson correlation.

## Results

The mean CAA and Schneider angle were 8.04^◦ ^(3.46) and 19^◦ ^(6.99), respectively (Table 1). 

The Pearson correlation analysis found significant positive correlation between S and CAA (r=0.826, P<0.001).

The mean radius, length, height and distance of canal curvatures are presented in Table 1. Negative correlations were found between radius and length (r= –0.4, P<0.001), radius and S (r= –0.4, P<0.0001), radius and CAA (r= –0.24, P=0.004) and CAA and y (r= –0.4, P<0.001). 

There was no correlation between height and Y (r=0.013, P=0.789), and CAA and height (r=0.654, P=0.001).

## Discussion

Root canal morphology and the degree of curvature are important factors for success in different stages of endodontic treatment.

Long, narrow and curved canals are most prone to transportation during instrumentation. Instrument deformation and fracture in curved root canals can cause serious problems during root canal therapy. 

Access cavity preparation, biomechanical efficacy of endodontic irrigants and obturation of the root canal system can all be influenced by root canal curvature.

To improve clinical success of endodontic treatments and allow easier comparison between various investigations on curved root canals, a thorough knowledge of root canal morphology is essential; canal curvature should also be precisely measured and described. Most studies on curved root canals have only used the Schneider angle. 

Only a few recent studies have used the Schneider angle in combination with the radius of curvature (10,11,6). 

However, using Schneider angle with the radius of curvature will only depict the apical geometry of root canals curves and not the coronal part of the root canal. 

CAA together with height and distance of curvature provide more information about the coronal geometry of root canal curvatures. 

Canals that are measured in this way may have different abruptness of curvature in the apical part of the canal. Thus, the shape of root canal curve is more accurately described using two angles, Schneider in combination with CAA and the related parameters including radius, length, distance and height of curvature. This combination provides more accurate guidelines for both coronal and apical parts of canal curvature.

In the present study, we measured root canal curvature on scanned radiographs using a computerized program. A buccolingual approach was used; K-files size #10 were carefully inserted into the canals avoiding any aberration in canal direction, similar to that described by Cunninghum (12) and Weine (13). However, all root canals have curvatures in the mesiodistal direction which cannot be seen in a routine radiography or buccolingual view. Furthermore, the silver point, or file, may not confirm the exact canal shape, as they do not remain centered in the canal lumen (1,5). Although this technique can not measure the exact curvatures of root canals, it is possible to determine the curve with which the instrument has to negotiate in order to reach the apical end of the canal (1,5).

No previous study has measured root canal curvature using both Schneider and canal access angle together with radius, length, height and distance of curvature; however, our results were relatively similar to that reported by Gunday *et al.* (9). In their study, the mean values of S and CAA were reported 22.42^◦ ^(6.31) and 15.45^◦ ^(4.99), respectively. They also found a positive correlation between S and CAA (r=0.093, P<0.001), CAA and canal length (r=0.31, P<0.001), CAA and height (r=0.74, P<0.001) and a negative correlation between CAA and curvature distance (r= -0.38, P<0.001).

## Conclusion

We can conclude that only precise guidelines can determine root canal curvature. Schneider and CAA angles together with radius, length, distance and height of curvature may be considered as a precise guideline.
